# Management and Outcomes of Osteochondritis Dissecans of the Knee in the Pediatric and Adolescent Population

**DOI:** 10.1007/s12178-026-10040-z

**Published:** 2026-06-06

**Authors:** Samuel I. Rosenberg, Nikhil B. Patel, Eileen A. Crawford

**Affiliations:** Department of Orthopedic Surgery, University of Michigan, 24 Frank Lloyd Wright Drive, Ann Arbor, MI 48106-0391 USA

**Keywords:** Osteochondritis dissecans, OCD, Cartilage, Osteochondral allograft, Osteochondral autograft transfer, Autologous cartilage implantation

## Abstract

**Purpose of Review:**

Osteochondritis dissecans (OCD) of the knee is a complex condition in pediatric and adolescent patients, and management relies heavily on lesion characteristics including size, stability, and fragmentation. The purpose of this review is to inform readers of the current understanding, treatment options, and outcomes of the disorder in pediatric and adolescent patients.

**Recent Findings:**

While management of OCD of the knee is tailored highly to patient- and lesion-specific factors, broad management pathways are dictated first by lesion stability, and further by salvageability of unstable lesions. Lesion appearance on MRI and arthroscopy are critical in informing appropriate management. Stable lesions, especially in skeletally immature patients, should undergo 3-6 months of nonoperative management, with an initial 4-6 week period of nonweightbearing and avoidance of high impact or repetitive stress for a minimum of 3 months. Patients with stable lesions who fail nonoperative treatment are candidates for retro- or trans-articular drilling of the lesion to promote healing. Unstable lesions may be amenable to salvage approaches including arthroscopic or open fixation with metallic screws, bioabsorbable implants, autograft, and suture bridge constructs. When not salvageable, osteochondral autograft transfer, osteochondral allograft transplantation, or autologous chondrocyte implantation is recommended and pursued depending on lesion size and subchondral involvement. All techniques have good potential for success in healing, patient reported outcomes, and return to activity when applied in the appropriate circumstances.

**Summary:**

Significant heterogeneity exists in management and outcomes of OCD of the knee, largely attributed to the varying presentation and treatment modalities. Management pathway should be patient-specific, however there is a paucity of robust comparative trials within specific populations.

## Introduction

### Overview

Osteochondritis dissecans (OCD) of the knee in pediatric and adolescent patients is a focus of ongoing research on a condition without a known definitive etiology. OCD lesions exist along a spectrum ranging from stable, intact lesions to unstable fragments and loose bodies. Lesion stability remains the primary determinant of both management strategy and prognosis.

Osteochondritis dissecans was first described in 1870 by Franz König, who characterized the condition as a process of spontaneous necrosis leading to separation of an osteochondral fragment [1]. Since then, advances in imaging and histologic evaluation have refined our understanding of the bone-cartilage interface and the proposed multifactorial pathophysiology of these lesions. Within the knee, OCD lesions most commonly occur in the medial femoral condyle (MFC, approximately 63.6%–85%), followed by the lateral femoral condyle (LFC, 15–32.5%), patella (1.5–10%), and trochlea (2%).^2^

Repetitive microtrauma remains the leading etiologic theory [2]. At the onset, premature regression of epiphyseal vessels causes epiphyseal cartilage necrosis, resulting in *osteochondrosis latens *[3, 4] This damaged cartilage does not initially involve the articular cartilage or subchondral bone, but as the ossification front spreads towards the lesion, endochondral ossification does not occur in the lesion, and the remnant of necrotic epiphyseal cartilage remains in the subchondral bone (*osteochondrosis manifesta) *[4] These lesions can self-resolve, however, may progress to *osteochondrosis dissecans* if subjected to repetitive microtrauma [5]. Lesions of the MFC may be related to repetitive impingement from a prominent medial tibial eminence during knee flexion and athletic activity, leading to cumulative subchondral stress. The MFC has been shown to have a relatively limited intraosseous blood supply with possible watershed areas [6]. Disruption of the blood supply in these areas at the articular-epiphyseal cartilage complex have been proposed as a contributing factor in OCD pathogenesis, supporting the role of vascular insufficiency in OCD development [7]. Additional contributing factors likely include genetic predisposition and abnormal ossification. Moderate obesity has also been associated with an increased risk of knee OCD [8].

### Classification

Numerous classification systems exist based on radiographic, magnetic resonance imaging (MRI), and arthroscopic findings [9]. While each provides value, all ultimately aim to distinguish stable from unstable lesions, which guides treatment. In stable OCD lesions, the subchondral bone and overlying cartilage remain fused to the surrounding bone and cartilage. Instability is charactized by a separation of the lesion from the parent bone. Radiographically, the Berndt and Harty classification was originally developed to describe talus lesions but has been extrapolated to be used for knee OCD lesions [10]. The radiographic system uses plain radiographs to describe a stepwise progression of lesions from subchondral compression (Stage 1) to loose body formation (Stage 4). The De Smet classification was one of the first systems to identify the presence of a high signal intensity line or cyst beneath the OCD lesion, cartilage fracture, or surface defect as being the most predictive criteria of instability on MRI [11]. Arthroscopic classification remains the gold standard for assessing stability due to direct visualization and probing [9]. The most commonly used is the International Cartilage Repair Society (ICRS) classification, which has four overarching grades of the lesions based on stability/discontinuity with arthroscopic probing [12]. Each grade is further divided into two subgroups, with “A” representing lesions less than 10 mm deep and “B” for lesions greater than 10 mm deep, enabling classification of both stability and size. Most recently, the Research in OsteoChondritis dissecans of the Knee (ROCK) group established an arthroscopic classification system split into two subgroups of immobile and mobile lesions [13]. The lesions are then further separated based on the visual appearance of the lesion (i, e, cue ball, wrinkle in the rug, trap door). This classification system has demonstrated great inter-observer and intra-observer reliability of 0.95 and 0.96, respectively [13].

While arthroscopy provides direct assessment of the cartilage surface, it does not fully evaluate subchondral pathology, and there may be an under-interpretation by arthroscopy for this reason [14]. In analogy to that of an iceberg, the surface of the lesion may appear stable, but underlying findings such as cysts at the border of the lesion may demonstrate impending instability [14]. Thus, a combination of MRI and arthroscopic evaluation can provide a more complete understanding of the lesion stability. The Dipaola classification integrates both modalities, helping to delineate between the middle classification tiers that have a similar arthroscopic appearance but span the borderline between stable and unstable [15]. A summary of key features of the various classification systems can be found in Table 1.

**Table 1:** OCD Classification Systems

Classification System	Imaging Modality	Classification Stages			
		Stable		Unstable	
Berndt and Hardy^10^	Radiographs	I: Subchondral compression fracture	IIa: Chondral fracture (partial fracture) IIb: Subchondral cyst	III: Chondral fracture with separated segment (non-displaced)	IV: Chondral fracture with separated segment (displaced)
DeSmet^9^	MRI	Criteria to predict lesion instability: ● High T2 signal intensity line surrounding the lesion ● Fluid-filled cartilage defect ● Presence of subchondral cysts ● High T2-signal fracture line in articular cartilage			
International Cartilage Repair Society (ICRS)^12^	Arthroscopy	1: Stable with continuity; intact overlying cartilage 2: Partial continuity; stable on probing		3: Complete discontinuity; not dislocated 4: Dislocated fragment	
		Size Identifier: A = < 10 mm, B = > 10 mm			
Research in OsteoChondritis dissecans of the Knee (ROCK)^13^	Arthroscopy	1 (Cue Ball): No abnormality detectable arthroscopically 2 (Shadow): Cartilage is intact and subtly demarcated 3 (Wrinkle in the Rug): Cartilage is demarcated with a fissure, buckle, and/or wrinkle		4 (Locked Door): Cartilage fissure at periphery, unable to hinge open 5 (Trap Door): Cartilage fissuring at periphery, able to hinge open 6 (Crater): Exposed subchondral bone defect	
Dipaola^15^	MRI + Arthroscopy	Stage I: MRI - Thickening of articular cartilage and low signal changes Arthroscopic - Irregularity and softening of articular cartilage; no definable fragment. Stage II: MRI - Articular cartilage breached; low signal rim behind lesion Arthroscopic - Articular cartilage breached; definable non-displaced fragment		Stage III: MRI - Articular cartilage breached; high signal changes behind fragment Arthroscopic - Articular cartilage breached; displaceable but attached fragment Stage IV: MRI - Loose body Arthroscopic - Loose body	

A table of commonly used OCD classification systems and their associated imaging modalities, as well as key staging characteristics

OCD = osteochondritis dissecans; MRI = magnetic resonance imaging

### Imaging

Plain radiographs are the first-line imaging modality for diagnosis and lesion localization; however, early or stable lesions may be radiographically occult. Standard views should include weight-bearing anteroposterior (AP) and lateral radiographs, in addition to a notch (tunnel) view to assess the posterior femoral condyles (PFC) and a sunrise view for evaluation of the patellofemoral joint. Since bilateral OCD occurs in approximately 15–30% of pediatric patients, radiographs of the contralateral knee are often obtained to assess for asymptomatic lesions [16–18]. Younger patients may also present with normal variants of ossification, particularly in the PFC, which mimic OCD lesions [19]. Contralateral films can help differentiate symmetric developmental variants from early OCD pathology. MRI is the preferred advanced imaging modality, as it allows for both detection and assessment of lesion stability. Criteria for obtaining MRI are for patients with equivocal radiographs with persistent pain, large lesions that require stability grading (surface area greater than 350 mm^2^ is used as a threshold by some authors [20]), closed physes, or symptoms greater than 6 months [20]. T2-weighted sequences typically demonstrate hyperintensity corresponding to subchondral edema.^21^ High-intensity signal between the progeny fragment and the surrounding bone is the most specific for instability [21]. Precise measurement of lesion dimensions (length, width, and depth) is critical for guiding management decisions and preoperative planning. However, the specificity of MRI is lower in patients with open physes [22]. Other imaging modalities, such as bone scintigraphy and computed tomography (CT), are additional options for lesion assessment and preoperative planning but are less frequently used due to increased radiation exposure and limited additional diagnostic utility compared to MRI [20].

## Treatment of Stable Lesions

### Nonoperative Management

Management is first stratified by lesion stability, as it is widely accepted that skeletally immature individuals with stable lesions experience favorable outcomes with nonoperative management [23–25]. There are multiple nomograms used to assist with predictive tasks on healing potentials [26, 27]. Notably, the ROCK research group has implemented a machine learning classifier to estimate which lesions are more amenable to non-operative healing [28]. Their findings noted that lesion location in the posterior aspect of the condyle and medial-most or lateral-most locations (away from the primary load-bearing surfaces) were associated with higher success. As lesions increased in width relative to condylar width, there was a lower likelihood of nonoperative success.

First-line management consists of activity modification - avoiding activities that cause repetitive and compressive stress loading, such as running, jumping, and squatting. The American Academy of Orthopaedic Surgeons (AAOS) does not recommend a single protocol, but supports 3–6 months of activity restriction with or without weight-bearing limitations or immobilization [23, 29]. High-impact and shear activities should be avoided for a minimum of 3 months from diagnosis until the patient’s pain has resolved and there is radiographic evidence of healing. Some providers will allow patients to participate in low-impact exercise such as swimming or riding a bike to maintain physical fitness. Notably, a gold standard for evaluation and determination of radiographic healing is controversial, with full radiographic healing often not apparent for greater than one year [23, 30, 31]. The degree of radiographic healing needed to safely initiate return to higher impact sports is not yet well-established. This is an important area of future research as clinicians do not currently have clear evidence-based guidelines on when to return patients to sports once their symptoms have resolved but the OCD lesion is still radiographically visible.

### Immobilization and Bracing

The role of immobilization remains debated. Casting may improve compliance with activity restriction, particularly in younger patients, and can be molded to offload affected compartments [14, 26]. However, casting limits knee motion and subsequently decreases joint surface synovial fluid nutrition, while leading to stiffness and muscle atrophy. While literature demonstrates the benefits of synovial fluid nutrition for unstable lesions with healing cartilage, there has been no evidence that this provides a benefit in stable lesions with intact cartilage coverage undergoing non-operative management. An alternative to casting is the use of an unloader brace, which provides stabilization of the knee and can alleviate excess stress to the MFC and LFC with valgus and varus unloading, respectively, while allowing for greater mobilization [14]. Despite the theoretical benefit of offloading a compartment with the presence of a lesion, a study by Tepolt et al. (2020) showed a higher rate of treatment failure with a single hinge unloader brace compared to crutches (50% vs. 35%; *p* < 0.02) [32] Wall et al. thus recommend the use of a dual hinge unloader brace due to its durability, as mentioned in their published protocol, which starts with 6 weeks of casting, followed by a dual hinge unloader brace and progressive rehabilitation [14]. While there is no pharmacologic management known to help with treatment of OCD lesions, emerging data suggest a potential association between vitamin D deficiency and OCD in adolescents, though causality remains unclear. Given low risk, screening and supplementation may be considered [33–35]. A 2019 systematic review outlined various non-operative methods including restriction of physical activity, physio- and kinesiotherapy, muscle strengthening exercises, physical instrumental therapies (iontophoresis and extracorporeal shock wave therapy), weight-bearing limitations, and immobilization. They found no single superior nonoperative protocol, though activity restriction consistently demonstrated favorable outcomes and had a higher healing rate [36, 37].

Overall, outcomes regarding nonoperative management remain variable, with some studies reporting patients with stable lesions to have a greater than 80% chance of healing,^25^ whereas a prospective protocol study identified roughly only two-thirds of patients responded to non-operative management and healed within 6 months of treatment [27]. A systematic review of recent nonoperative studies reported an overall healing rate of 61.4%, however it highlights significant heterogeneity in the included studies that may contribute to variability in reported healing rates [36]. Additionally, this variability in healing rate likely reflects the other factors besides stability that can impact success with nonoperative treatment, including physeal status, lesion location, coronal plate alignment, and adherence to restrictions.

### Follow-up and Rehabilitation

**Fig. 1:**
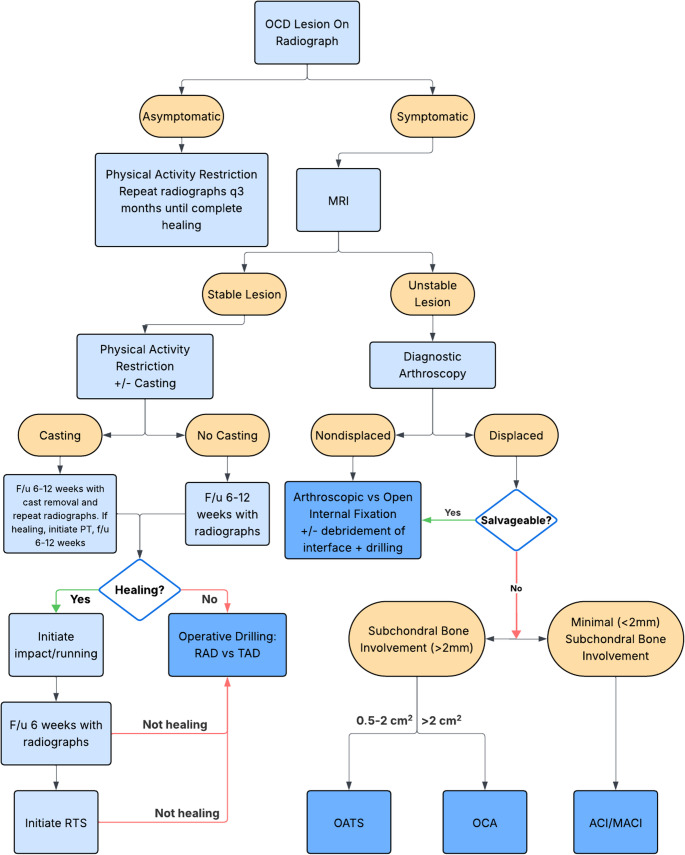
Flowchart demonstrating authors’ preferred management algorithm for OCD lesions of the child and adolescent knee. OCD = osteochondritis dissecans; PRN = as needed; MRI = magnetic resonance imaging; F/u = follow up; PT = physical therapy; RTS = return to sport; RAD = retrograde articular drilling; TAD = transarticular drilling; OATS = osteochondral autograft transfer; OCA = osteochondral allograft transplantation; ACI = autologous chondrocyte implantation; MACI = matrix-induced autologous chondrocyte implantation

While current literature places a heavy emphasis on thorough trials of nonoperative management, there has been a paucity in literature since 2019 regarding a standard protocol for monitoring and return to activities. In the absence of a standard of care, the senior author’s approach is described in this paragraph. Nonoperatively managed patients follow up every 6–8 weeks to report symptoms, have the knee examined for concerning signs, and obtain repeat radiographs to assess for signs of healing. If casting is used, the cast should be removed after 6 weeks, and physical therapy should be initiated to regain range of motion and quadriceps function. In the absence of casting, this interval can be lengthened to 3 months to limit radiation exposure with serial radiographs, particularly at the initiation of restrictions. Once symptoms resolve, patients are typically anxious to return to activities, and the shorter interval allows for a timelier progression of activities if radiographic healing is observed. Criteria used at our institution for beginning a return to higher impact activity such as running include: (1) at least 3 months of restriction from high impact activity, (2) resolution of symptoms, and (3) evidence of at least partial radiographic healing in the form of decreased lesion size, osseous consolidation, or boundary resolution. Physical therapy can be prescribed to ensure gradual progression through a running and strengthening program with good body mechanics. Patients and their caregivers should be given strict instructions that the return of symptoms with these higher impact activities are a sign to stop the activity and contact their treatment team for further guidance. Our patients return for another evaluation with radiographs 6 weeks after initiation of running. If the patient remains asymptomatic without concerning physical exam findings and the radiographs show further healing, the patient is then allowed to progress back to competitive sports participation. If there is no change in the radiographic appearance of the OCD lesion, the period of restriction may be extended, and if the lesion appears worse, a discussion of surgical intervention is warranted. Once the patient has completely returned to full activity without restriction, follow-up is recommended anytime symptoms recur. Additionally, a lesion that is not completely healed by the time of full clearance should be monitored approximately every 6 months until full radiographic healing is observed. Even after initial clinical and radiographic healing is observed, some patients with stable OCD lesions will have progression of the disease that warrants surgical intervention [38]. A graphic of our preferred management strategy for OCD lesions, both nonoperative and operative, can be seen in Fig. 1.

### Operative Management for Stable Lesions

**Fig. 2:**
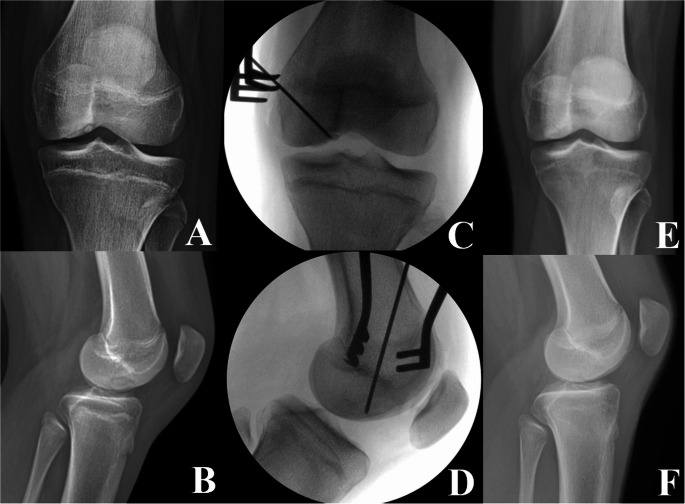
Retroarticular drilling for a stable medial femoral condyle lesion of a left knee. (A) and (B) demonstrate preoperative anteroposterior and lateral radiographs, respectively. (C) and (D) demonstrate intraoperative drilling using a start point below the physis. Anteroposterior and lateral radiographs at 9 months postoperative can be seen in (E) and (F), respectively

Surgical intervention is considered after at least 6 months of failed nonoperative management or earlier in select cases of stable lesions with poor prognostic factors. For stable lesions, drilling techniques are recommended to stimulate healing [23]. Two major techniques are transarticular drilling (TAD) and retroarticular drilling (RAD), which promote healing by causing increased blood supply and recruiting biologic factors from underlying cancellous bone at the site of the lesion. This is typically performed with multiple passes of a 0.045-inch (1.1 mm) or 0.062-inch (1.6 mm) Kirschner wire across the lesion boundary [23, 24]. TAD involves drilling through the articular cartilage into the lesion under arthroscopic visualization. Advantages include direct visualization, reduced reliance on fluoroscopy, and shorter operative and tourniquet time [39]. The RAD approach, on the other hand, involves drilling from outside the joint into the lesion, preserving the articular surface [39]. An example of RAD for a stable MFC lesion can be seen in Fig. 2. Advocates for the RAD method argue that it allows for access to more posterior lesions and optimizes healing of the articular surface without the direct violation that occurs via the TAD approach, however it requires intra-operative fluoroscopy and can be technically challenging. Additionally, stable lesions with fully intact cartilage may not be visible arthroscopically for a TAD approach, whereas fluoroscopic guidance with RAD technique ensures that drilling spans the entire lesion. However, the ROCK study performed arthroscopy in the RAD group and noted that in 22% of RAD cases, there was at least one iatrogenic perforation of the articular surface [40]. A number of studies have investigated healing after treatment of stable lesions with either RAD or TAD, with a systematic review by Gunton et al. reported healing rates to be similar (86% and 91% at 5.6 and 4.5 months for RAD and TAD, respectively). More recently, a randomized controlled trial compared RAD and TAD for treatment of stable OCD in 91 skeletally immature knees found that TAD was associated with a shorter surgical time, less fluoroscopy use, and a quicker return to sport with improved patient-reported outcomes and superior healing on radiographs at 6- and 12-month follow-up, but found no difference in such parameters at 24-month follow-up.^40^ Therefore, both techniques have acceptable longer-term outcomes and the technique utilized typically depends on surgeon preference.

## Operative Management of Unstable Lesions

In contrast to stable lesions, unstable lesions often require initial operative intervention without a trial of conservative measures. Historically, treatment with loose body removal and debridement was thought to lead to acceptable short-term outcomes; [41–43]however, more recent literature has suggested a high percentage of these patients experience early degenerative joint disease [44]. A 2017 systematic review performed by Sanders et al. demonstrated patients who underwent fragment excision experienced a higher rate of osteoarthritis and conversion to total knee arthroplasty at a mean follow-up of 16 years when compared to those undergoing cartilage preservation or grafting procedures [45]. As such, management of unstable lesions is generally divided into repair and restoration modalities for salvageable and unsalvageable lesions, respectively.

### Salvageable Lesions

**Fig. 3:**
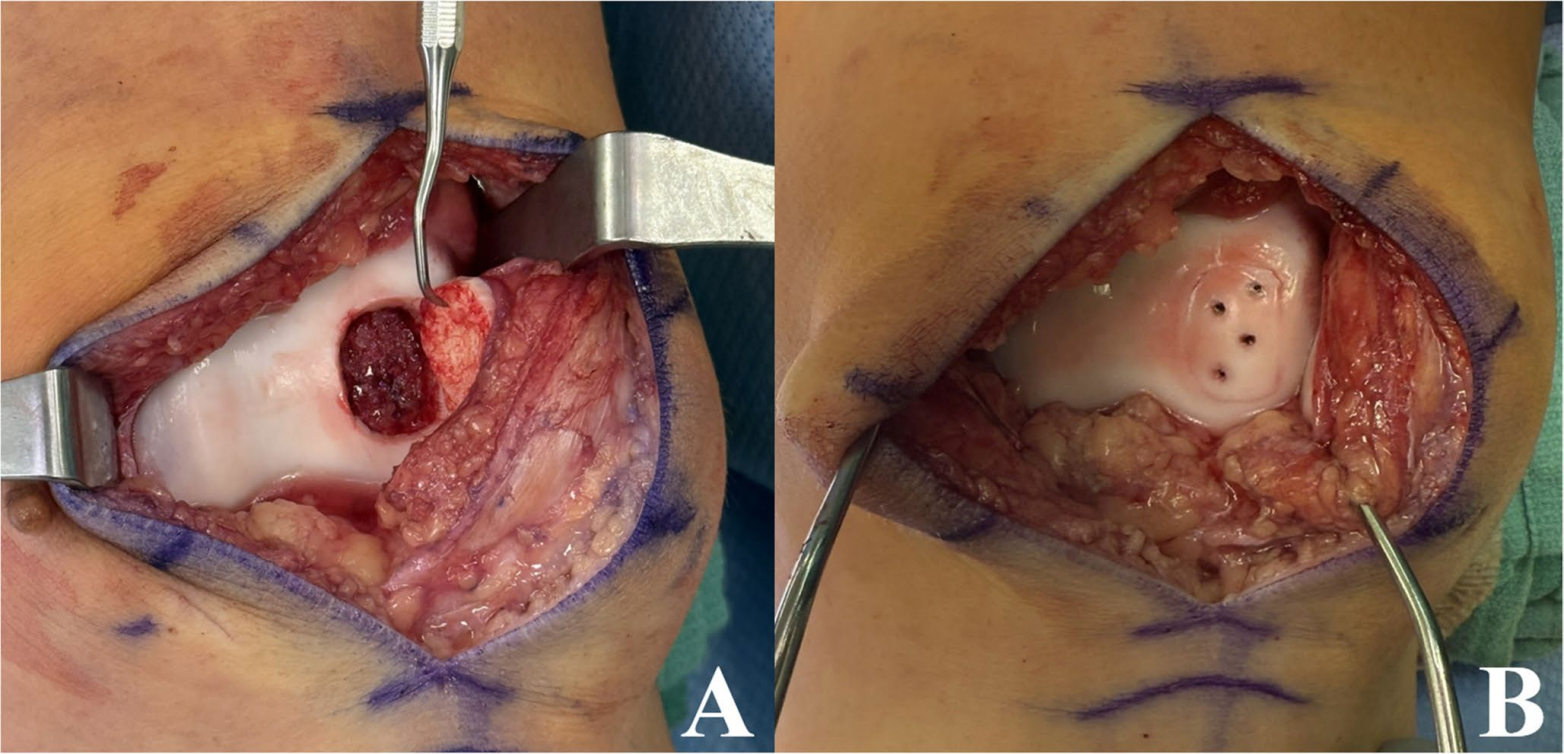
Open reduction and internal fixation of a trochlear lesion using four metallic (titanium) screws. (A) demonstrates debridement of the underlying host/progeny interface while hinging the lesion open, while (B) demonstrates the reduced lesion with screws in place

When an unstable OCD lesion is deemed salvageable (i.e. limited fragmentation, reduced or reducible lesions without significant cartilage degeneration), repair can be performed via a multitude of techniques, including arthroscopic or open fixation with metallic screws, bioabsorbable or biologic implants, and suture bridge constructs. Concomitant fibrous debridement,^24^ drilling/microfracture,[24, 46, 47] and/or bone grafting [48] are often performed at the time of fixation to supplement the repair and improve biologic healing. Metallic screws are historically common and provide durable fixation, however generally require a second procedure for removal to prevent iatrogenic cartilage damage [24, 49–52]. Headless metallic screws can be left in place if buried beneath the cartilage, though some surgeons choose to remove them after 4–5 months of healing time due to the potential for significant damage if the lesion fails to heal and breaks free, leaving an exposed screw. An example of open reduction and internal fixation of a trochlear lesion using metallic screws can be seen in Fig. 3. Bioabsorbable implants forego the need for hardware removal, but can break, lose fixation, and often promote a foreign body reaction with resultant chronic joint effusion and cyst formation [24, 53]. A 2020 study of 58 children and adolescents undergoing fixation with biodegradable nails exemplifies this with 6-month postoperative MRIs demonstrating broken nails and persistent effusions in 37% and 39% of patients [54]. Other studies show less concern, with a recent 2024 study of 13 patients undergoing OCD fixation with hydroxyapatite poly-L-lactic acid (HA/PLLA) threaded pins finding only 1 patient to experience nonunion secondary to nail breakage at 24 month follow up [55]. Fixation with autograft, such as autologous bone pegs, also avoids the need for hardware removal while limiting concern for foreign body reactions, and provide enhanced healing due to the use of autograft; however, robust data supporting this methodology is limited [56–58]. More recently, suture bridge constructs have been utilized for fixation of unstable lesions given their ability to provide variable compression and versatility to treat all-cartilage progeny, or lesions with fragmented subchondral bone [59–63]. Early supporting data has been favorable, with a 2023 study investigating 14 knees with OCD lesions (mean age 15.1 years, lesion size 362 mm^2^) to experience full union in 9 out of 14 patients and stable union in 5 of 14 patients on MRI at mean 16 months postoperatively, with improvement in KOOS scores at 6 and 12 months without any reported failures [63]. Given the variable methods for fixation, systematic reviews of this patient population are rather heterogenous. However, a 2023 review of 81 skeletally immature and mature patients with unstable OCD lesions who underwent either open or arthroscopic internal fixation by Husen et al. found a healing rate of 72% at mean follow up of 11.3 years, with failure defined as need for further surgical management [64]. They noted skeletal maturity had no significant correlation to failure rate; however, lesions in the lateral femoral condyle were found to be at higher risk for failure.

### Unsalvageable Lesions

**Fig. 4:**
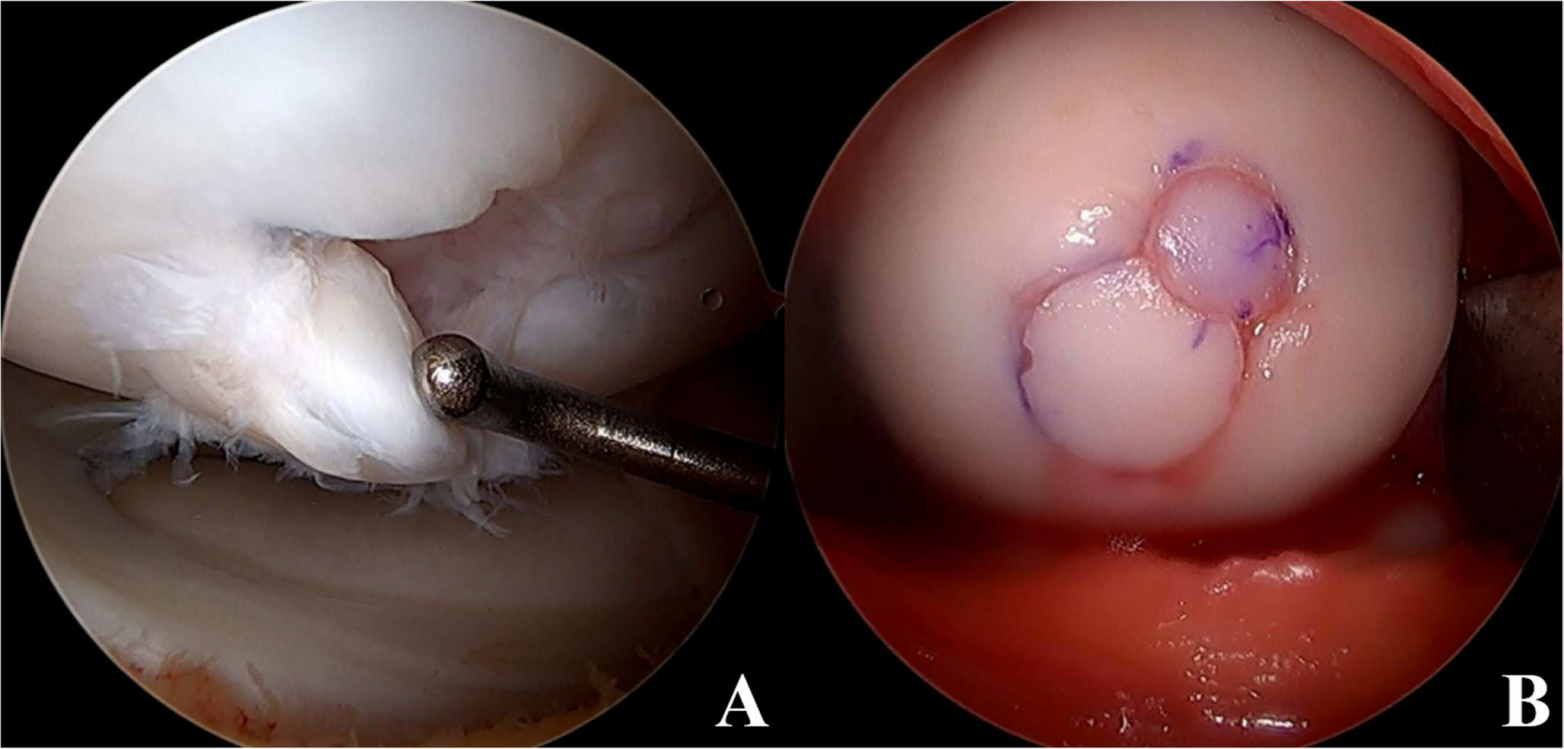
Osteochondral autograft transfer (OATS) of a lateral femoral condyle lesion. (**A**) demonstrates arthroscopic evaluation of an unstable, unsalvageable osteochondral dissecans lesion and (**B**) showing OATS using two cartilage plugs from the ipsilateral trochlea

The most common techniques to address unsalvageable lesions (i.e. fragmented, deteriorated chondral surface, irreducible) include osteochondral autograft transfer (OATS), osteochondral allograft transplantation (OCA), and autologous chondrocyte implantation (ACI) or matrix-induced autologous chondrocyte implantation (MACI). OATS and OCA are thought to be the best options for lesions with significant subchondral bone involvement, with general guidelines deeming OATS useful in cases of 0.5–3 cm [2] and OCA in defects greater than 3cm [2] or in revision cases [23] Given OATS includes autograft harvesting, donor graft is usually obtained from limited weightbearing portions of the ipsilateral femoral condyle, with recommendation of obtaining approximately 10 mm of subchondral bone for press fit fixation in congruent or < 1 mm countersunk fashion [23]. Utilization of OATS for treatment of an unstable, unsalvageable LFC lesion can be seen in Fig. 4. OATS procedures have demonstrated significantly better results when compared to microfracture, with a 2009 randomized controlled trial of 50 patients with mean OCD lesions approximately 3.2 cm [2] finding 81% versus 14% of patients returning to preinjury levels in OATS versus microfracture groups, respectively [65]. Although not just in pediatric patients, a 2024 investigation by Orazi et al. found favorable long-term results of OATS in 20 patients with OCD lesions and mean 12.6 year follow up, with no reported overall failures, and with smaller lesions (< 2 cm [2]) and lesions of the MFC performing better overall in regard to patient reported outcome measures (PROMs) [66].

**Fig. 5:**
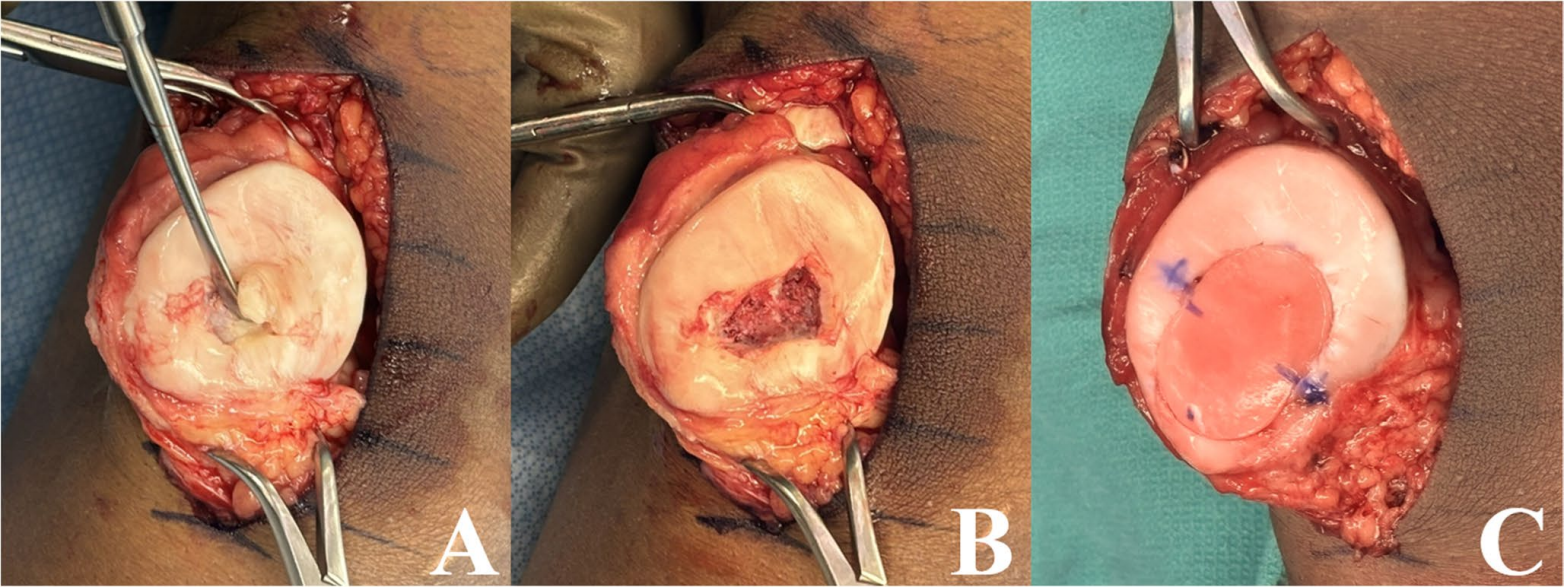
Osteochondral allograft (OCA) transplantation of a patellar lesion, with the lesion depicted in (**A**), and again after debridement to subchondral bone in (**B**). The final construct with implanted OCA plug from a donor patella can be seen in (C)

**Fig. 6:**
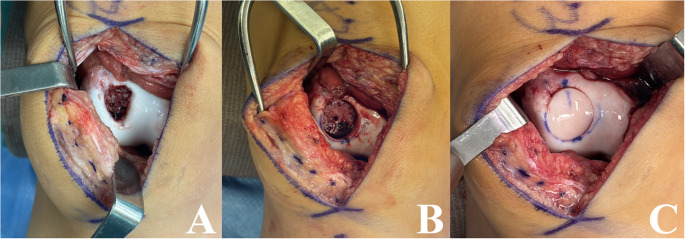
Osteochondral allograft (OCA) transplantation of a large trochlear lesion, with the lesion depicted in (A) and again after coring the recipient site for transplantation in (B). (C) demonstrates the final construct after implantation of donor trochlear plug

OCA requires donor-recipient size matching, prolonged wait times, high cost, and need for fresh, refrigerated specimens (with freezing leading to inadequate viability) [67]. Examples of OCA for treatment of patellar and trochlear lesions can be seen in Figs. 5 and 6, respectively. Prior literature has demonstrated improved PROMs, high rate of graft incorporation, and high satisfaction and return to activities at both short- and long-term follow up, and in revision settings in children and adolescents with unstable cartilage lesions [68–71]While prior investigations have demonstrated success in both skeletally immature and mature patients,^69^ Gilat et al. found that patients with closed physes showed greater improvement in PROMs than those with open physes at mean 4.6 years follow up, without a difference in reoperation or failure rate between groups [72].

Alternative to the use of osteochondral auto- or allografts, ACI is a two-stage technique that utilizes cell culturing techniques to cultivate chondrocytes using a patient’s own chondral tissue. In an index procedure (often alongside diagnostic arthroscopy of a known lesion), a cartilage biopsy from a non-weightbearing portion of their femoral condyle (i.e. intercondylar notch or trochlea) is obtained and sent to a laboratory for processing and growth over a 2–4 week period [23, 24]. In the second stage, the lesion is debrided to a stable base, and the cultivated cells are re-implanted under a membrane at the site of the defect. Matrix-induced autologous cartilage implantation (MACI^®^) is a modification of this procedure, during which the biopsied chondrocytes are impregnated into a porcine collagen membrane matrix over 4–6 weeks prior to implantation, allowing for implantation in a single unit instead of under a biologic matrix [23, 24]. ACI procedures have been recommended for lesions from 2 to 16 cm^2^ with overall favorable clinical outcomes in long-term follow up in multiple studies, including both children and adults [24] Although including both children and adults (mean age 26.1, range 14–52 years), a recent study from Sweden with median follow up of 19 years demonstrated an overall low graft failure rate of 13% at 10 years, 15% at 15 years, and 18% at 20 years [73]. It is important to note that recommendations indicate ACI/MACI techniques only when there is less than 8 mm of subchondral bone loss, given the procedure involves re-implantation of chondrocytes alone. However, recent literature has proposed several techniques that combine ACI/MACI procedures with autologous bone grafting to build back the underlying subchondral bone [74, 75]. This may prove to still be insufficient in larger lesions > 3–4 cm [2], with worse outcomes and persistent abnormalities found in subchondral bone on postoperative MRIs [75, 76]. Similarly, A 2021 comparative study by Paatela et al. of 115 patients with either full-thickness cartilage lesions or OCD lesions undergoing ACI found that patients with OCD lesions had a significantly higher failure rate than those with full-thickness cartilage lesions without subchondral involvement (43.3% vs. 19.1% at 10 year follow up) [77]. In the authors’ opinion, these cartilage restoration procedures should be used sparingly for the treatment of OCD, and only when the diseased bone that is inherent to OCD can be reliably replaced as part of the procedure.

### Patient Outcomes and Recommendations

Overall, given the heterogeneity of patient characteristics (skeletal maturity, mechanical alignment, and activity level) and lesion characteristics as well as surgical techniques, there is a lack of a consensus on proposed treatment algorithms. Much of the existing literature is clouded by the fact that the etiology of cartilage lesions (i.e. traumatic versus OCD) is often grouped or undistinguished in the published literature, which is noteworthy given the distinct pathophysiology of OCD lesions. Within this limitation, prior systematic reviews have demonstrated OATS, OCA, and ACI/MACI all provide favorable clinical outcomes with the ability to achieve minimal clinically important difference thresholds when treating cartilage lesions [78].

In our practice, unsalvageable OCD lesions are most often treated with OCA for large lesions and OATS for lesions that are small enough to be treated with 1–2 osteochondral plugs. OCD lesions are ultimately a disorder of the subchondral bone, so a complete removal of the osseous portion of the lesion and replacement with a structural graft is prioritized.

## Return to Play

Especially in the pediatric patient, discussion of return to impact and return to play is paramount to the recovery process in both nonoperative and operative management. Objective measures for return to activities are challenging, as lesions do not always follow a predictable time course of healing, and a gold standard for evaluation of radiographic healing of OCD lesions remains controversial [30, 31]. As with much of the OCD-related literature, heterogenous definitions and postoperative protocols limit cross-study comparability [79]. Within that context, a 2025 systematic review of return to sport (RTS) of patients with stable OCD lesions (13 articles, 783 knees) noted 85–100% and 100% rates of return to sport, ranging from 3.7 to 8.1 and 2.8 to 8.5 months for nonoperative and operatively managed lesions, respectively [80]. Regarding unstable lesions, results comparing techniques are largely heterogenous, however it has repeatedly been demonstrated that patients who undergo microfracture procedure routinely suffer from decreased RTS rates than those undergoing OCA, OATS, and ACI/MACI procedures [81–84]. RTS rates and timelines specific to the OCD population remain highly variable and inconclusive, subject to the variability in lesion size, location, and stability. A 2024 systematic review of 168 patients (ages 15–25) found RTS percentages to be 64.5% for MACI, 76.5% for OATS, and 94% for internal fixation with a variable mean return to sport timeline from 6.5 to 9.7 months,^83^ however others have proposed timelines nearing 9 to 16 months for these procedures [84]. Most importantly, patient and caregiver expectations should be managed from the time of diagnosis. There is a large range of healing time regardless of technique, and failure of one treatment path will necessitate starting over with a new treatment.

## Conclusions

There is wide variability in pathways of both nonoperative and operative management of children and adolescents with OCD lesions of the knee. Generally, stable lesions have a high likelihood of success with nonoperative management and can be further managed with TAD versus RAD when nonoperative modalities are unsuccessful or in patients with poor prognostic indicators. Management of unstable lesions relies heavily on preserving and optimizing patients’ own cartilage, when possible (i.e. fixation), versus restoring healthy hyaline cartilage or osteochondral units when necessary. Although a complex pathology with risk for progression and failure, optimizing treatment modalities regarding patient- and lesion-specific factors maximizes healing, patient reported outcomes, and return to activity and sport. Future high-quality, comparative, and multi-centered research is critical to providing evidence-based guidelines that improve management, outcomes, and counseling for these young patients.

## Key References

- Gilat R, Haunschild ED, Huddleston H, Parvaresh KC, Chahla J, Yanke AB, Cole BJ (2021) Osteochondral Allograft Transplantation of the Knee in Adolescent Patients and the Effect of Physeal Closure. Arthroscopy 37:1588–1596.
  - ○ A retrospective comparative study of adolescents undergoing OCA for large OCD, demonstrating similar PROs and reoperation in patients 16 years and younger compared to 17-18 year old patients, however noting patients with closed physes to experience a greater increase in PROs after OCA.
- Heyworth BE, Ganley TJ, Liotta ES, et al (2023) Transarticular Versus Retroarticular Drilling of Stable Osteochondritis Dissecans of the Knee: A Prospective Multicenter Randomized Controlled Trial by the ROCK Group. Am J Sports Med 51:1392–1402.
  - ○ A multicenter randomized controlled trial which demonstrates that both TAD and RAD have favorable healing rates for stable OCD lesions in skeletally immature patients, with TAD having superior healing at 6 and 12 months, but no difference between the two groups at the 24-month mark.
- Husen M, Van der Weiden GS, Custers RJH, Poudel K, Stuart MJ, Krych AJ, Saris DBF (2023) Internal Fixation of Unstable Osteochondritis Dissecans of the Knee: Long-term Outcomes in Skeletally Immature and Mature Patients. Am J Sports Med 51:1403–1413.
  - ○ A larger, retrospective multicenter study demonstrating healing rate of 72% at a mean follow-up of 11.3 years of unstable OCD lesions after internal fixation.
- Johnstone T, Espiritu J, Tompkins M, et al (2024) Which Osteochondritis Dissecans Lesions Will Heal Nonoperatively? An Application of Machine Learning to the ROCK Prospective Cohort. Orthop J Sports Med 12:23259671241297145.
  - ○ A case-control study utilizing machine learning to identify predictors of improved healing success with non-operative management of OCD lesions in skeletally immature patients, including lesion location and lesion size.
- Metz AK, Riederer M, Gagnier J, Crawford EA (2022) Incidence of Subsequent Surgical Intervention at Short-term Follow-up in Previously Healing and Stable Juvenile Osteochondritis Dissecans of the Knee. J Pediatr Orthop 42:e271–e276.
  - ○ A single-center, retrospective review of skeletally immature patients with stable OCD lesions initially managed nonoperatively, demonstrating that although the majority of patients have favorable outcomes with nonoperative management, some may progress to surgical intervention after returning to activity.
- Muchintala R, Coladonato C, Perez A, Kellish A, Mumtaz S, Sutton W, Wilson S, Cohen S, Tjoumakaris FP, Freedman KB (2025) Return to Sport After Treatment of Stable Osteochondritis Dissecans Lesions of the Knee in Adolescents: A Systematic Review. Am J Sports Med 53:1761–1768.
  - ○ A recent systematic review of patients 18 years and younger undergoing nonoperative and operative treatment of OCD, demonstrating favorable return to sport rates with heterogenous treatment modalities and timelines.

## Data Availability

No datasets were generated or analysed during the current study.
